# The effect of different colored light emitting diode illumination on egg laying performance, egg qualities, blood hormone levels and behavior patterns in Brown Tsaiya duck

**DOI:** 10.5713/ab.20.0657

**Published:** 2021-01-14

**Authors:** Chin-Hui Su, Chih-Hsiang Cheng, Jung-Hsin Lin, Hsiu-Chou Liu, Yen-Ting Yu, Chai-Ching Lin, Wei-Jung Chen

**Affiliations:** 1 Department of Biotechnology and Animal Science, National Ilan University, Yilan, Yilan 260007, Taiwan; 2 Ilan Branch, Livestock Research Institute, Wujie Township, Yilan 268020, Taiwan

**Keywords:** Brown Tsaiya Duck, Illumination, Light Emitting Diode

## Abstract

**Objective:**

The objective of this experiment was to investigate the effects of different colors produced by light emitting diode (LED) on Brown Tsaiya ducks.

**Methods:**

A total of 144 female Brown Tsaiya ducks were randomly allocated into three individual cage rearing chambers with different LED illumination colors as treatments. Three different treatments were: i) white color, ii) blue color, and iii) red color. The experiment periods were from ducks 21 to 49 weeks of age, determined traits included i) egg laying performance, ii) feed intake, iii) egg shell breaking strength, iv) egg shell thickness, v) egg Haugh unit, vi) egg weight, vii) serum Estradiol and Progesterone concentration, and viii) behavior pattern.

**Results:**

The results indicated that when compared with white and blue color, red color could stimulate ducks sexual maturation and raised the egg laying performance. The red light group was also observed to have the highest feed intake among three treatments. The blue treatment had the lowest egg shell breaking strength and the highest egg weight among three treatments, nevertheless, no significant difference was observed among three treatments on egg shell thickness and egg Haugh unit. The red light group had higher serum estradiol concentration than the white and blue groups, but no significant difference among treatments on the serum Progesterone concentration was found. The results of behavior pattern indicated that red light group showed more feeding and less resting behavior compared to the blue light group.

**Conclusion:**

We found a potential of applying red light illumination in the indoor laying duck raising system with positive results on egg laying performance and acceptable egg weight, equivalent egg qualities compared to white and blue light.

## INTRODUCTION

Environmental manipulation is an effective means to improve poultry production and welfare [1,2]. Among those environmental factors, light plays a vital role in affecting chicken production [3]. Light has five basic characteristics: source, intensity, duration, uniformity, and wavelength (light color) [4]. Research on poultry lighting dates back to the early 1930s. Since then, extensive research has led to a broad understanding of lighting effects on poultry [5]. Recently, more energy-efficient, durable, affordable, and dimmable light-emitting diode (LED) lights are increasingly finding applications in poultry production. Due to light is a crucial environmental factor that affects bird behavior, development, production performance, health, and well-being [6,7], the emerging LED lighting in poultry housing has getting increasing attention from both scientific and industrial communities.

Because of having specific adaptations, like a fourth type of retinal cone and eye lenses that are clear to short-wave light [8,9], poultry can see a wider range of the light spectrum than humans. The estimation of their visual range differs somewhat between studies, starting around 350 to 380 nm and ending at 760 to 780 nm [9]. This means that poultry’s visual spectrum includes UV-A light, but likely just excludes infrared light. This wide spectrum was not only determined by assessing the electrophysiological capacity of the cone cells but also confirmed using behavior tests [8].

It is found that laying hens were healthier and showed better laying performance with 25% less cholesterol in the egg when they were given whole spectrum illumination [10], another reference compared the effect of fluorescent tube and LED on laying hens found that no significant influence on laying performance and 7% of feed intake and 17% of energy cost was reduced in LED treatment [11]. Nonetheless, some review article concluded that there was no influence on laying performance when given white and green illumination stimulation on laying hens [12].

Tai and Hsieh [13] compared the effects of five different wavelengths LED illumination including blue (473 nm), green (530 nm), red (625 nm), orange (603 nm), and warm white (5500 K) on broiler breeding eggs’ hatchability, the results indicated that eggs exposure blue, red and warm white illumination showed better hatchability, however, green light increase the deformity rate.

Raising ducks on the floor with litter and water pond in the field is the conventional method to breed ducks in Taiwan. However, with the level of invasion of avian influenza getting severer, it is a trend to raise ducks in the duck house. Because ducks accept natural illumination when they are bred by conventional methods, an artificial illumination source has to be a supplement in the duck house that ducks can receive enough lighting stimulation. Most of the illumination used in Taiwan in animal husbandry houses is still fluorescent tubes and high-pressure sodium lamp. They are more inefficient compare to the LED on lifetime and energy cost. The advantages of LEDs are high energy change efficiency, small volume, long lifetime, fixed wavelength, and low heat. LED illumination has high energy savings potential. There is also no mercury in LED lamps, contributing less to environmental pollution. Some researches focused on the LED illumination effects on poultry. However, only a few references tested this effect on waterfowl. Therefore, this experiment investigates the effects of different colors produced by LEDs on Brown Tsaiya ducks. This study sought to find the best illumination pattern for indoor laying ducks.

## MATERIALS AND METHODS

### Animal care

The experiment was conducted between February and September 2015 and carried out at the Ilan Branch, Livestock Research Institute, Council of Agriculture, Taiwan (24°40′22″ N and 121°49′55″ E, and altitude of 7 m above sea level). The experimental procedure was approved by the Institutional Animal Care and Use Committee at Ilan Branch, Livestock Research Institute, Council of Agriculture (case number: 104 - 007).

### Experimental light

**Figure 1:**
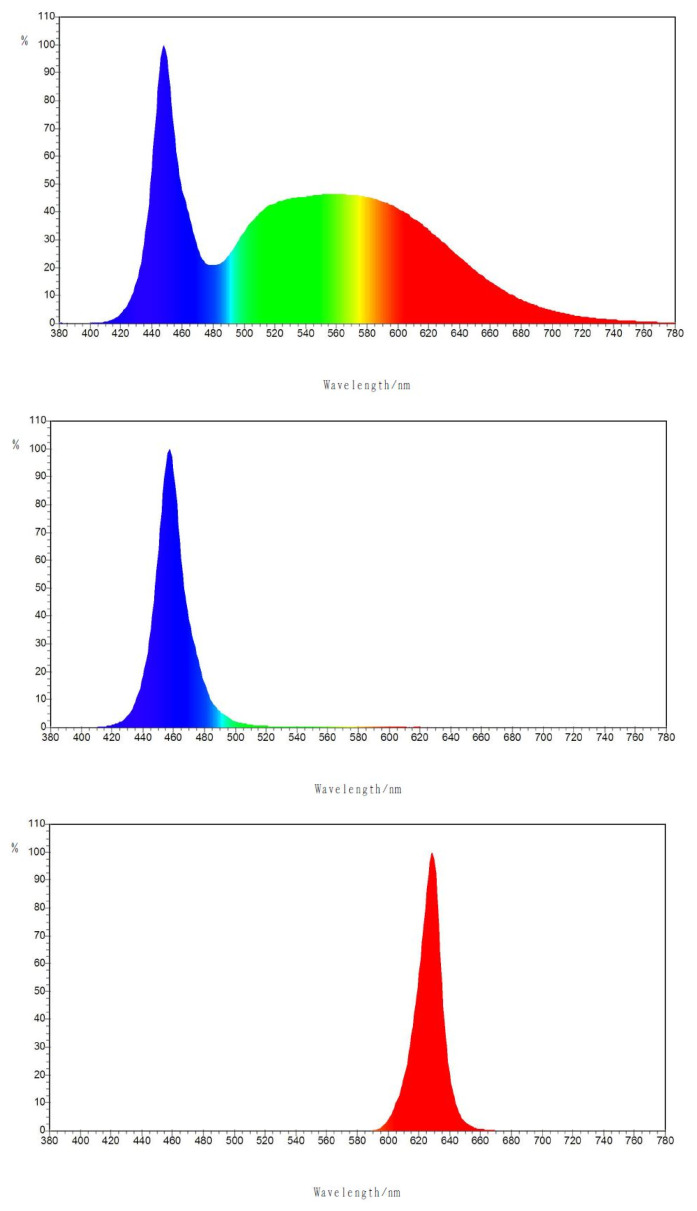
The wave length distribution of the white, blue and red light emitting diode (LED) lamp in the experiment. The peak wavelengths for blue and red light were 630 and 460 nm, determined by Integrating Sphere, Zvision, Beijing, China.

The experimental LED (Sanan Optoelectronics Co. Ltd., Xiamen, China) illumination was placed at the ducks’ head level. The average light intensity was 60 lux based on blue light. White and red lights were adjusted following Benoit [14] as similar light stimulation. The daily lighting pattern was 16 L:8 D and the white light color temperature was 6000K. The peak wavelengths for blue and red light were 460 and 630 nm (wavelengths distribution were shown in Figure 1, determined by Integrating Sphere, Zvision, Beijing, China), respectively. Ducks were given a 16 L:8 D lighting program with the illumination time from 5 am to 9 pm. The dark time was from 9 pm to 5 am.

### Experimental animal

One hundred forty-four self-bred female brown Tsaiya ducks were used in this experiment. All ducks were reared in a breeding house and fed starter diets until 4 weeks of age. Thereafter, ducks were moved into a semi-open duck house and fed a grower diet during the growing period until 18 weeks of age. At 18 weeks of age, the ducks were randomly allocated into three individual opaque chambers with layer diets given at the same time. After three weeks of layer diet, cage feeding and experimental illumination were adapted. The experiment was started at 21 weeks of age and lasted to 49 weeks of age.

### Experimental diet

Ducks were provided with *ad libitum* commercial duck starter diet from hatching to 8 weeks of age. Grower and layer diets were provided between 8 to 18 weeks of age and after 18 weeks, respectively. Different diet compositions and calculated nutrition levels are shown in Table 1. Food and water were given *ad libitum* during this experiment.

### Experimental facility

Three identical opaque chambers, each measuring 570×330× 235 cm (L×W×H), were used in the laying phase. Each chamber contained 48 cages (12 cages per tier×4 tiers), with each measuring 40×30×45 cm (D×W×H) with a space allowance of 1,200 cm^2^/duck. The feeder was hung outside the front side of the cage. A one nipple drinker was set on the backside. The thermal environment conditions in the chambers were controlled using two diameter 40.64 cm fans in the front and backside of the chamber, respectively.

### Experimental method

#### Egg laying performance

The number of eggs laid was recorded daily for egg production calculation. Feed intake was collected by calculating the sum of residual feed in 4 tiers as 4 repeats on 3 consecutive days during the week.

#### Egg quality

All of the eggs were collected for egg quality determination, including egg weight, egg shell breaking strength, egg shell thickness, egg albumen height, Haugh unit on three consecutive days biweekly and tested within 24 h after collection. The experimental procedures are described below:

- Egg weight: an electronic scale (FAY-06, Nagata Inc., Tainan, Taiwan) was used for egg weight determination.
- Egg shell breaking strength: put the point side up, then crack the eggs with an egg shell break instrument (HT-9635A, Hung Ta Inc., Taoyuan, Taiwan) and record the maximum breaking force.
- Egg shell thickness: three different parts, including the point side, blunt side and the central portion of the eggs were collected from each egg for egg shell thickness determination (FN595, FHK Inc., Tokyo, Japan). The recorded egg shell thickness values were the average of three different measurements.
- Egg albumen height: egg albumen height was measured using a tester (FHK Egg Quality Gauge, FHK Inc., Japan)

#### Blood hormone levels

Sixteen fixed ducks in the middle area of each treatment were chosen for duck blood Estradiol and Progesterone concentration, analyzed biweekly after 22 weeks of age. Between 2 and 3 mL of blood were collected using syringes from the wing vein of each duck. The blood was injected into a plasma separation gel and lithium heparin vacutainer (BD 367871, BD, Franklin Lakes, NJ, USA) and temporarily stored on an ice bath. After all samples were collected, the vacutainers were spun down in an IEC MULTI-RF220v centrifuge for 15 min at 3,000 rpm to separate the cells from the plasma. The plasma was poured into 2 mL microcentrifuge tubes and stored at −18°C until further analysis. Plasma Estradiol and progesterone concentration determination were entrusted to a commercial analytical laboratory (Health Medical Laboratory) using commercially available kits (06656021 190; 07092539 190, Roche, Basel, Switzerland) according to the manufacturer’s instructions.

#### Animal behavior observation

From 21 weeks of age, every two weeks, ducks were recorded using a digital video recorder (HDR CX-240, Sony, Tokyo, Japan) positioned in the front part of each chamber. Video recording was used four days in a week for behavior observation in each treatment until 49 weeks of age. Five ducks were observed for 8 hours on an observation day with 4 observation days in a week. The first observation time was 30 minutes after recording begin to avoid worker disruption. At each hour, ducks were monitored for 15 seconds for behavior determination. Behavior observation during the experiment was visually recorded and analyzed by one observer to minimize behavior definition variation induced by different observers. Duck behavior definition was followed and slightly modified from Lee [15]. Duck behaviors were divided into three main categories: feeding (including feeding and drinking), activities (including preening, frolicking and wagging) and resting (including standing, crouching and sleeping). The behavior definitions are described in Table 2.

### Statistical analysis

Statistical analyses were performed using SAS enterprise (SAS enterprise guide 7.1, SAS institute, Inc., Cary, NC, USA). All variables were analyzed using the general linear model procedure in a completely random design with different colored LEDs as the main effects. The differences among treatments were evaluated using Tukey’s honestly significant difference. Probability values of <0.05 were taken to indicate significance. Duck behavior patterns were subjected into three categories including feeding, activities and resting. These three categories were analyzed with the categorical data analysis (CATMOD) procedure to compare the difference between the three treatments.

## RESULTS

### Egg laying performance

The egg laying performance results for all treatments are shown in Table 3. The results indicated that the red LED treatment had significantly higher egg laying performance for the testing period of 27 to 32 (84%±2%), 39 to 44 (71%±4%) weeks of age and the whole testing period (74%±9%)(p<0.05). There were no significant differences between the white and blue LED treatments for the most part in the testing period except for the 39 to 44 weeks of age period.

### Feed intake

The feed intake results represent higher egg laying performance for the red LED treatment (Table 4). In the 21 to 25 (157±15 g), 27 to 31 (151±6 g), 45 to 49 (162±8 g) weeks of age and whole testing period (153±12 g). The red light group had the highest feed intake of all groups. This may combine with their better egg laying performance. There was no significant difference between the white and blue LED treatments throughout the whole experimental period.

### Egg quality

The white LED treatment had the highest egg shell breaking strength throughout the whole experiment except for the first 4 weeks (Table 5). The red light treatment showed no significant difference with the blue light in most of the testing period. However, the red light treatment ducks laid eggs with significantly higher egg shell breaking strength compared to the blue light throughout the whole experiment (5.25±0.45 vs 5.08±0.49 kg/cm^2^). There were some significant differences between treatments during the 21 to 25 (blue vs white) and 33 to 37 (white vs red) weeks of age. There was no significant difference in egg shell thickness and egg Haugh unit between treatments throughout the whole experiment (Tables 6, 7). The eggs laid by the blue LED treatment had heavier egg weight during the 27 to 49 weeks of age period and therefore caused heavier egg weight (64.6±4.4 g) than eggs laid by the white (63.1±4.0 g) and red (62.8±4.5 g) LED treatments through the whole experiment (Table 8).

### Blood hormone levels

Ducks exposed to the red LED illumination showed higher blood estradiol at 22 to 26 (421±168 pg/mL), 34 to 38 (442± 304 pg/mL) weeks of age and therefore, through the whole experiment (red 471±296 pg/mL vs white 344±356 pg/mL and blue 336±188 pg/mL). However, the blood progesterone concentration results between different treatments did not show any significant difference in this experiment (Table 9).

### Behavior observation

As shown in Table 10, ducks exposed to blue light significantly change their behavior compared to the white (p = 0.0223) and the red illumination (p = 0.0352). There was no significant difference in behavior between white and red treatments.

## DISCUSSION

From the egg laying performance results, we found that the red LED illumination stimulated the duck sexual maturation. The red LED light treatment showed a significantly higher egg laying performance trend from 21 to 26 weeks of age (p = 0.0664) and determined periods thereafter (Table 3). This is consistent with the results of Borille et al [16] who found that white and red LED treatment ducks laid more eggs than blue, yellow and green LED treatments when given 17 hours of illumination. Pyrzak et al [17] also reported that red light stimuli hens produced more eggs than blue light in two laying cycles. Kim et al [18] concluded that pullets under red LED sexually matured earlier than those in all other light treatments including white and blue LED light. Blue LED light also delayed the hens’ sexual maturation time compared to incandescent light (control), white and red light. Our experiment observed that red LED treatment laid more eggs from 21 to 32 weeks of age. Hassan et al [19] tested the effects of different illuminations including red, green, blue, white, combined red, green and red, green, blue. Their results indicated that there were no significant differences between the treatments which contained at least 12 hours of red light. This showed the importance of red light on laying hens reproductive traits.

It is already known that birds had peak sensitivity to yel low and green wavelengths. Nevertheless, some references indicated that birds exposed to green light would delay sexual maturation time, exhibit reduced egg production and GnRH-I mRNA expression [20,21]. Furthermore, the green light treatment would inhibit reproduction [20]. In contrast, some researches demonstrated that red lights could accelerate sexual development and maturity in poultry [20,22]. The results from some studies showed that monochromatic red light produced higher egg production than those reared under white, green [17,21–23] and blue LED light [24].

Our results for the effects of different color illumination on feed intake (Table 4) were not in agreement with some researches [11,16,24,25]. Our results showed that red light treatment had similar feed intake with white light. However, the experiment result by Kim et al [18] indicated that laying hens exposed under red light showed higher feed intake than white and blue light. The feed intake difference in our experiment may partially represent the higher egg production from red LED treatment and hence there were no significant differences between white and blue treatments for the most part in this experiment. The feed intakes between these two groups were similar.

Some references referred that exposure to short wave length lights (e.g., green and blue color) led to improved egg quality (e.g., egg weight, shell thickness or shell strength) when compared to long wavelength lights (e. g., red color) [17,19]. However, the improved egg qualities in these cited studies were associated with the relatively lower egg production of birds as reported in the studies to a certain extent. In contrast, many cited studies reported that no differences between or among lights in sexual maturity or egg production performance of birds [16,20,24–26]. The different lighting sources were also found to have no effect on egg qualities. Broille et al [16] found that the internal egg qualities (albumen height, specific gravity, and Haugh units) of ISA brown hens at 56 to 72 weeks of age were not influenced by the illumination source including incandescent light, blue, yellow, green, red or white LED light. Kamanli et al [26] found that incandescent, fluorescent or LED lights did not influence egg qualities. The recent study from Liu et al [5] also indicated that there were no significant differences between poultry-specific LED light and warm-white fluorescent light. Archer [25] did not illustrate a difference in egg production or quality between red and white LED light bulbs. The egg weight result was consistent with Pyrzak et al [17], who reported that hens exposed to blue light laid eggs heavier than eggs laid by hens exposed to red light. Hassan et al [24] reported that red light and white light had the same level of egg weights but both of them were lighter than eggs laid by blue light treatment. In contrast to Svobodova et al [23] referred that there was no significant difference between blue, green, red and yellow light treatments on ISA brown egg weight. Archer [25] reported that red and white LED illumination did not change the egg weight of white Leghorn hens after 54 weeks of treatment. Our results suggest that red light stimulation made lighter egg weight than blue ones. Instead of the higher egg shell breaking strength, if the lighter duck eggs weights are acceptable in the commercial market, given ducks red light would be a better choice for higher egg laying performance and egg shell breaking strength with no adverse effects on egg shell thickness and Haugh unit (Tables 5 to 8).

Some references indicated that the different wavelength illumination could affect the behavior of animals. For meat type chicken, Sultana et al [28] found that broilers were shown to spend more time sitting or standing under short wavelengths (blue/green) and exhibited more locomotion under longer (red/yellow) wavelengths. Prayitno et al [27] concluded that meat chickens in the red and white light were more active, which was evident in the white-reared birds in greater walking activity and in the red-reared birds in greater floor pecking, wing stretching, and aggression. However, this stimulation pattern is not suitable for egg type chicken. Huber-Eicher et al [22] reported that the green light (short wavelength light) stimulated laying hens spent more time foraging and pecking at objects than the red light, in contrast, red light increased laying hens feeding frequency, indicating the different light stimulation effects on different poultry species. In our experiment, ducks exposed under red LED illumination showed more time spent on feeding, partially agreed with it, however, owing to the breed (chicken vs duck) and rearing system (floor vs cage) difference, further study should be done for clarifying the actual color stimulation pattern on caged laying ducks.

The duck’s plasma estradiol concentration result (Table 9) was partially in agreement with Baxter et al [21] who compared red, green and white light stimulation on white leghorn laying hens. He found that at 20 weeks of age the Estradiol concentrations of the red treatment were significantly higher than those of white and blue treatments. Hassan et al [24] also found that laying hens 22 weeks of age after 10 weeks of red light exposure showed higher serum estradiol concentration than blue light groups. Higher levels of estradiol during egg laying initiation have been correlated with the activity of small follicles [29]. It may partially explain the higher egg laying performance of the red treatment in our experiment. Although the role of estradiol and progesterone are to develop the reproductive organs and initiate ovulation [30], we could not find any difference between the treatments on plasma progesterone. This may be because the oviposition time of ducks occurs from 10 pm to 4 am and blood sample collection time is about 9 am, 13 to 19 hours before oviposition. Thus, the progesterone concentration was at a relatively low level in the serum [30] and thus made the progesterone result difficult to observe.

## Figures and Tables

**Table 1:** Diet ingredients and composition in the rearing period (0 to 8 weeks of age), growing period (8 to 18 weeks of age) and laying period (after 18 weeks of age)

Items	Rearing diet	Growing diet	Layer diet
Ingredients			
Yellow corn	55.54	51.94	49.93
Soybean meal (44%)	25.30	10.00	27.00
Wheat flour middling’s	10.30	20.00	-
Wheat bran	-	10.00	6.50
Fish meal	2.00	-	3.30
Yeast powder	3.00	2.00	2.00
Rice hull powder	-	2.40	-
Soybean oil	1.10	-	2.50
Limestone	1.10	1.60	6.60
Di-calcium phosphate	1.10	1.50	1.50
Salt, iodized	0.30	0.30	0.40
Choline chloride (50%)	0.08	0.08	0.08
Lysine-HCl	-	-	0.01
DL-methionine	0.05	0.05	0.05
Vitamin premix	0.03	0.03	0.03
Mineral premix	0.10	0.10	0.10
Calculated nutritional value			
ME (kcal/kg)	2,900	2,660	2,700
Crude protein (%)	19.5	13.5	20.0
Calcium (%)	0.81	0.94	3.05
Available phosphorus (%)	0.36	0.44	0.44
Methionine (%)	0.38	0.27	0.39
Lysine (%)	1.05	0.60	1.11

ME, metabolizable energy.

**Table 2:** The definition of cage rearing Brown Tsaiya ducks behaviors

Behavior	Definition
Feeding	Pecking feed
Drinking	Pecking water supplier
Preening	Touching the feather via beak or scratching via claw
Frolicking	Shaking head rhythmically or pecking the cage, nearby ducks’ body and head genetically, or head out of the cage
Wagging	Standing and wagging tail rapidly without other frolicking behavior
Standing	Standing with two feet, eyes looking at forward or around
Crouching	Duck’s breast and abdomen touching the cage bottom, feet crouched, eyes looking at forward or around
Sleeping	Leaning head on or inside the wings, duck stopped without moving

**Table 3:** The egg laying performance of Brown Tsaiya ducks in different treatment groups

Weeks of age	White LED	Blue LED	Red LED
	--------------Egg laying performance (%)---------------		
21 to 26	54±15	46±17	69±14
27 to 32	73±5^b^	77±3^b^	84±2^a^
33 to 38	70±3	76±4	71±6
39 to 44	63±5^b^	71±3^a^	71±4^a^
45 to 49	69±2	68±7	74±2
Means	66±10^b^	68±14^ab^	74±9^a^

Means±standard deviation.

LED, light emitting diode.

a,b Means in the same row without a common superscript differ significantly (p<0.05).

**Table 4:** The feed intake of Brown Tsaiya ducks in different treatment groups

Weeks of age	White LED	Blue LED	Red LED
	-------------- Feed intake (g/d/bird) -------------		
21 to 25	145±17^ab^	140±18^b^	157±15^a^
27 to 31	140±7^b^	145±8^ab^	151±6^a^
33 to 37	142±12	151±8	143±13
39 to 43	145±14	152±8	153±7
45 to 49	155±15^ab^	151±12^b^	162±8^a^
Means	145±14^b^	148±12^ab^	153±12^a^

Means±standard deviation.

LED, light emitting diode.

a,b Means in the same row without a common superscript differ significantly (p<0.05).

**Table 5:** The egg shell breaking strength of Brown Tsaiya ducks in different treatment groups

Weeks of age	White LED	Blue LED	Red LED
	-------- Egg shell breaking strength (kg/cm^2^) --------		
21 to 25	5.29±0.54	5.05±0.63	5.33±0.51
27 to 31	5.59±0.45^a^	5.34±0.40^b^	5.47±0.40^ab^
33 to 37	5.46±0.38^a^	5.20±0.33^b^	5.37±0.44^ab^
39 to 43	5.35±0.45^a^	5.04±0.45^b^	5.06±0.28^b^
45 to 49	5.06±0.54^a^	4.76±0.42^b^	5.03±0.40^a^
Means	5.35±0.50^a^	5.08±0.49^b^	5.25±0.45^a^

Means±standard deviation.

LED, light emitting diode.

a,b Means in the same row without a common superscript differ significantly (p<0.05).

**Table 6:** The egg shell thickness of Brown Tsaiya ducks in different treatment groups

Weeks of age	White LED	Blue LED	Red LED
	-----------------Egg shell thickness (mm) ----------------		
21 to 25	0.399±0.016^b^	0.406±0.010^a^	0.401±0.013^ab^
27 to 31	0.400±0.009	0.395±0.010	0.398±0.010
33 to 37	0.398±0.009^a^	0.397±0.011^ab^	0.392±0.009^b^
39 to 43	0.400±0.014	0.399±0.014	0.398±0.011
45 to 49	0.382±0.014	0.382±0.014	0.383±0.015
Means	0.396±0.014	0.396±0.014	0.394±0.013

Means±standard deviation.

LED, light emitting diode.

a,b Means in the same row without a common superscript differ significantly (p<0.05).

**Table 7:** The egg Haugh unit of Brown Tsaiya ducks in different treatment groups

Weeks of age	White LED	Blue LED	Red LED
	-------------------------- Haugh unit ----------------------------		
21 to 25	88±7	90±7	90±6
27 to 31	87±6^ab^	88±8^a^	86±8^b^
33 to 37	84±7	85±9	86±8
39 to 43	86±9^ab^	84±9^b^	88±9^a^
45 to 49	88±8	87±8	86±9
Means	87±8	87±9	87±8

Means±standard deviation.

LED, light emitting diode.

a,b Means in the same row without a common superscript differ significantly (p<0.05).

**Table 8:** The egg weight of Brown Tsaiya ducks in different treatment groups

Weeks of age	White LED	Blue LED	Red LED
	------------------------- Egg weight (g) --------------------------		
21 to 25	57.2±4.1	58.4±4.3	56.2±4.3
27 to 31	62.8±1.3^ab^	63.3±1.5^a^	62.1±1.4^b^
33 to 37	64.1±2.0^b^	65.4±1.8^a^	63.3±2.4^b^
39 to 43	65.0±2.2^b^	66.7±2.0^a^	65.4±1.5^b^
45 to 49	66.3±2.1^b^	69.0±1.8^a^	67.1±1.5^b^
Means	63.1±4.0^b^	64.6±4.4^a^	62.8±4.5^b^

Means±standard deviation.

LED, light emitting diode.

a,b Means in the same row without a common superscript differ significantly (p<0.05).

**Table 9:** The blood hormone levels of Brown Tsaiya ducks in different treatment groups

Items	White LED	Blue LED	Red LED
	------------------- 22 to 26 wk of age ---------------		
Estradiol (pg/mL)	360±362^ab^	280±126^b^	421±168^a^
Progesterone (ng/mL)	0.43±0.49	0.44±0.27	0.50±0.28
	-------------------- 28 to 32 wk of age --------------		
Estradiol (pg/mL)	329±247	358±237	400±174
Progesterone (ng/mL)	0.38±0.25	0.42±0.33	0.36±0.16
	------------------- 34 to 38 wk of age ---------------		
Estradiol (pg/mL)	315±157^b^	379±212^ab^	442±304^a^
Progesterone (ng/mL)	0.31±0.17	0.40±0.30	0.33±0.24
	-------------------- 40 to 44 wk of age --------------		
Estradiol (pg/mL)	411±607	352±202	407±411
Progesterone (ng/mL)	0.31±0.22	0.51±0.55	0.38±0.56
	-------------------- 46 to 48 wk of age --------------		
Estradiol (pg/mL)	285±132	299±70	415±379
Progesterone (ng/mL)	0.23±0.16	0.31±0.18	0.38±0.41
	-------------------------- Means --------------------------		
Estradiol (pg/mL)	344±356^b^	336±188^b^	417±296^a^
Progesterone (ng/mL)	0.34±0.30	0.43±0.36	0.39±0.35

Means±standard deviation.

LED, light emitting diode.

a,b Means in the same row without a common superscript differ significantly (p<0.05).

**Table 10:** The results of Brown Tsaiya ducks behavior pattern in different treatment groups (%)

Items	White LED	Blue LED	Red LED
Original behavior			
Feeding	7.9	7.0	7.8
Drinking	11.3	10.3	12.6
Preening	30	24.5	26.6
Frolicking	9.5	12.4	10.6
Standing	26.3	33.5	25.6
Crouching	6.3	5.1	8.0
Sleeping	3.1	1.1	3.9
Wagging	5.8	6.1	4.9
Main behavior category			
Feeding	19.12	17.25	20.38
Activities	45.25	43.00	42.12
Resting	35.63	39.75	37.50
Probability of ChiSq		0.0162	
Analysis of contrast			
White vs blue		0.0223	
White vs red		0.1359	
Blue vs red		0.0352	

LED, light emitting diode.
